# ANGPTL4 Regulates Psoriasis *via* Modulating Hyperproliferation and Inflammation of Keratinocytes

**DOI:** 10.3389/fphar.2022.850967

**Published:** 2022-07-04

**Authors:** Yuyue Zuo, Lei Dai, Li Li, Yuqiong Huang, Xinxin Liu, Xin Liu, Xiaoru Duan, Su Jiang, Guo-Min Deng, Hongxiang Chen

**Affiliations:** ^1^ Department of Dermatology, Union Hospital, Tongji Medical College, Huazhong University of Science and Technology, Wuhan, China; ^2^ Division of Cardiology, Department of Internal Medicine, Tongji Hospital, Tongji Medical College, Huazhong University of Science and Technology, Wuhan, China; ^3^ Department of Rheumatology, Union Hospital, Tongji Medical College, Huazhong University of Science and Technology, Wuhan, China; ^4^ Department of Dermatology, Huazhong University of Science and Technology Union Shenzhen Hospital, Shenzhen, China

**Keywords:** psoriasis, weighted gene co-expression network analysis, ANGPTL4, proliferation, inflammation

## Abstract

**Background**: Psoriasis is characterized by keratinocyte proliferation and massive inflammatory leukocytes infiltration, affecting 0.14%–1.99% of the world’s population. Our aim was to identify novel potential therapeutic strategies for psoriasis.

**Methods**: Weighted gene co-expression network analysis (WGCNA) was performed to identify gene modules that were closely related to psoriasis based on the GSE30999 dataset, which contained expression data from 85 patients with moderate-to-severe psoriasis. Then, angiopoietin-like 4 (ANGPTL4), one of the most related hub genes, was selected for *in vitro* and *in vivo* functional assays. In our experiments, imiquimod (IMQ)-induced psoriasiform dermatitis in mice and human keratinocytes (HaCaT) cells were used to study the potential roles and mechanisms of ANGPTL4 in psoriasis.

**Results**: WGCNA analysis revealed the turquoise module was most correlated with psoriasis, and ANGPTL4 is one of the most related hub genes that significantly upregulated in psoriasis lesions compared with non-lesional skin. Consistent with the bioinformatic analysis, the expression of ANGPTL4 was significantly upregulated in IMQ-induced psoriasiform skin of mice. Exogenous recombinant ANGPLT4 protein treatment could promote the proliferation and induce the expression of inflammatory cytokines in HaCaTs, whereas silencing of ANGPTL4 effectively inhibited these effects. Then we demonstrated that recombinant ANGPTL4 protein exacerbated psoriasiform inflammation and epidermal hyperproliferation *in vivo*. Mechanismly, extracellular signal-regulated kinase 1/2 (ERK1/2) and signal transducer and activator of transcription 3 (STAT3) pathways were involved in ANGPTL4-mediated regulation of proliferation and inflammation.

**Conclusion**: We found ANGPTL4 was significantly increased in IMQ-induced psoriasiform skin of mice. ANGPTL4 could promote keratinocyte proliferation and inflammatory response *via* ERK1/2 and STAT3 dependent signaling pathways in psoriasis.

## Introduction

Psoriasis is a common, immune-mediated, chronic papulosquamous skin disease. The estimated prevalence rate of psoriasis varies from 0.14% in east Asia to 1.99% in Australasia (Parisi et al., 2020). Psoriasis is characterized by hyperproliferative keratinocytes, acanthosis, elongation of dermal capillary vessels, and infiltration of massive inflammatory leukocytes into the dermis and epidermis. The disease severely affects the patients’ quality of life. According to the different clinical manifestations of skin lesions, psoriasis is mainly classified as chronic plaque, psoriasis vulgaris, pustular psoriasis, guttate psoriasis, and erythrodermic psoriasis (Griffiths et al., 2021). Genetic studies have clarified IL-17 and IL-23 as essential drivers of psoriasis development over the past few years (Ghoreschi et al., 2021). Immune targeting of these inflammatory cytokines by biological therapies has achieved promising results in severe chronic plaque disease. However, the specific molecular pathogenesis of psoriasis development is not fully clear and psoriasis cannot be cured currently. The investigations of novel therapeutic strategies to cure psoriasis are still required.

During recent years, the expeditious development of high-throughput sequencing and microarray has enabled researchers to further explore novel biomarkers and therapeutic targets for psoriasis at the molecular level. Weighted gene co-expression network analysis (WGCNA) is a widely used systems biology method that can construct a scale-free gene co-expression network and extract gene co-expression modules and hub genes highly related to the phenotypic traits (Langfelder and Horvath, 2008). In the present study, we constructed WGCNA algorithm to identify hub genes related to psoriasis. Angiopoietin-like 4 (ANGPTL4), the candidate hub gene, was selected for subsequent *in vivo* and *in vitro* functional assays.

ANGPTL4 is a multifunctional secreted protein that is expressed in adipose tissue, liver, kidney, muscle, ovary, breast, skin, urinary system, and other tissues (Guo et al., 2014). Its expression is regulated by the nutritional and metabolic status of the organism (Hato et al., 2008). ANGPTL4 contains an N-terminal coiled-coil region that mediates covalent homo-oligomerization and a C-terminal fibrinogen-like domain. ANGPTL4 undergoes proteolytic processing and releases the C-terminal domain, which circulates as a monomer. The n-ANGPTL4 mediates ANGPTL4 oligomerization and binds to lipoprotein lipase to modulate lipoprotein metabolism. C-ANGPTL4 is known to be involved in several non-lipid related processes, including angiogenesis, inflammation, oxidative stress, vascular permeability and wound healing (Padua et al., 2008; Goh et al., 2010a; Goh et al., 2010b; Huang et al., 2011; Jung et al., 2020). However, the expression and regulatory mechanism of ANGPTL4 in psoriasis remain unclear. This study explored the regulation and potential impact of ANGPTL4 on psoriasis. We have detected increased levels of ANGPTL4 in both plasma and psoriasiform skin of imiquimod (IMQ)-induced mice. We found ANGPTL4 could promote proliferation and significantly upregulate the levels of pro-inflammatory factors in psoriasis. Our results suggested the potential use of therapeutic approaches targeting ANGPTL4 in psoriasis treatment.

## Materials and Methods

### Data Collection and Data Preprocessing

We downloaded the raw microarray data of GSE30999 from the Gene Expression Omnibus (GEO) database (http://www.ncbi.nlm.nih.gov/geo/). Dataset GSE30999 was performed on GPL570 [HG-U133_Plus_2] Affymetrix Human Genome U133 Plus 2.0 Array. The skin biopsy samples were collected from 85 patients with moderate-to-severe psoriasis without receiving active psoriasis therapy. They were all participated in ACCEPT, an IRB-approved Phase 3, multicenter, randomized trial. The gene expression profiling sub-study was conducted. The analysis results identified 4,175 probe-sets as being significantly modulated in psoriasis lesions (LS) compared with matched biopsies of non-lesional (NL) skin.

**FIGURE 1:**
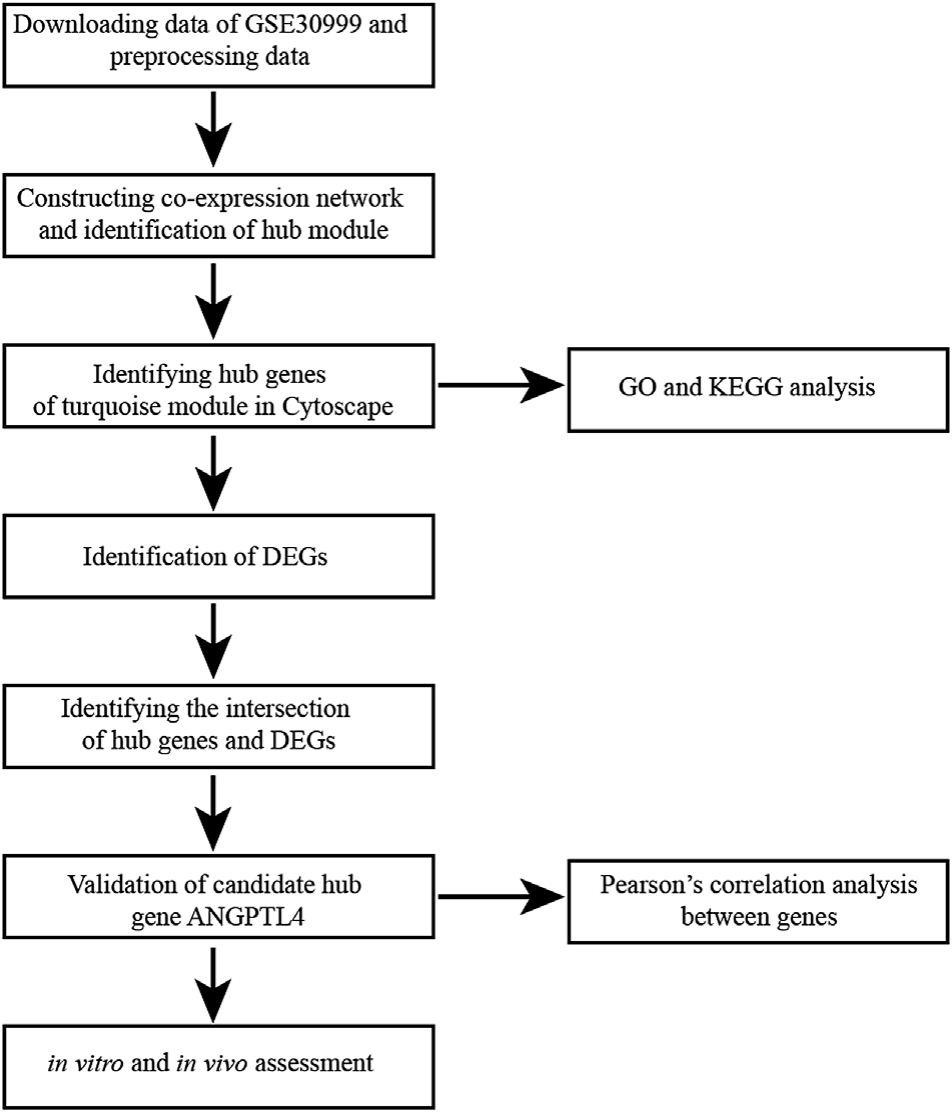
Flow diagram of data collection, preprocessing, analysis and validation in this study. KEGG, Kyoto Encyclopedia of Genes and Genomes; DEG, differentially expressed gene; GO, Gene Ontology.

We built a workflow for the process of the experiment (Figure 1). Under the R environment, the Robust Multi-array Average (RMA) algorithm in the “Affy” package was used to preprocess the raw data (excel files) (Gautier et al., 2004). After background correction, quantile normalization, probe summarization, and eliminating the unpaired data, we selected the top 25% of the most variant genes by analysis of variance for further analysis.

### Co-Expression Network Construction and Identification of Significant Modules

The R package “WGCNA” was used to conduct the co-expression network for the genes mentioned above (Langfelder and Horvath, 2008). Firstly, we constructed the weighted adjacency matrix with the formula a_mn_ = |c_mn_|^β^ (a_mn_ is adjacency between gene m and gene n, c_mn_ is Pearson’s correlation between gene m and gene n). The soft-thresholding parameter β was used to emphasize strong correlations and penalize weak correlations. Subsequently, we transformed the weighted adjacency matrix into a topological overlap matrix (TOM), which could measure the network connectivity of each gene defined as the sum of its adjacency with all other genes for network generation (Yip and Horvath, 2007). To classify genes with similar expression profiles into the same gene modules, we conducted the average linkage hierarchical clustering according to the TOM-based dissimilarity (1-TOM) measure with a minimum gene group size of 30 for the gene dendrogram (Ravasz et al., 2002). The branches of the tree representing highly correlated genes were grouped into one module and labeled with a specific color. Unassigned background genes that belonged to none of the modules were denoted with the grey section. The module eigengenes (MEs) and module significance (MS) were defined as previously described (Dai et al., 2021). We applied the MEs and MS to identify the significant module related to psoriasis.

### Hub Genes Identification and Validation

For the significant module, we uploaded the genes to the Search Tool for the Retrieval of Interacting Genes (STRING) database (Szklarczyk et al., 2021) and used the Cytoscape software to visualize the protein-protein interaction (PPI) network. To identify sub-networks of correlated genes, we used the “MCODE” plugins in Cytoscape (degree cutoff = 2, node score cutoff = 0.2, k-score = 2, and max. depth = 100). The “cytoHubba” plugins was applied to represent the top genes in the sub-networks. Then, we conducted “Limma” R package to recognize differentially expressed genes (DEGs) between the psoriasis lesions and non-lesional skin. |log_2_ fold change (FC)| ≥ 1.35 and adjusted *p*-value ≤ 0.05 were set for up-regulated and down-regulated genes. Volcano plot and heatmap were carried out by ggplot2 and pheatmap packages in R software. Venn diagram of the overlapping hub genes in the DEGs was drawn by jvenn web (http://jvenn.toulouse.inra.fr/app/example.html) (Bardou et al., 2014). We chose ANGPTL4 from the overlapped hub genes for further analysis and validated its expression and function in the following experiments.

### Functional and Pathway Enrichment Analyses

Database for Annotation, Visualization, and Integrated Discovery database (DAVID, https://david.ncifcrf.gov/) is an online analytical tool (Huang da et al., 2009b; a). All genes in the hub module were uploaded into DAVID to undergo Gene Ontology (GO), as well as Kyoto Encyclopedia of Genes and Genomes (KEGG) pathway enrichment analysis (Rue-Albrecht et al., 2016; Kanehisa et al., 2019). GO terms for biological processes (BP), cellular components (CC), and molecular functions (MF) were assessed, respectively. The cut-off criterion was *p* < 0.05.

### Animals

Female BALB/c mice (18–20 g) at 7 weeks old were purchased from Jiangsu Huachuang Sino Pharmaceutical Technology Co., Ltd. (Jiangsu, China). The mice were fed with standard laboratory food and water under controlled conditions (12 h: 12 h light-dark rhythm, 23 ± 2°C ambient temperature, 45–55% humidity). After being acclimatized for 7 days, the mice were randomly divided into several groups (*n* ≥ 5 per group). 62.5 mg daily topical dose of IMQ cream (5%, Mingxin Pharmaceuticals, Sichuan, China) were smeared on the dorsal shaved skin of mice for 7 consecutive days. Vaseline jelly was used as the control. The recombinant human ANGPTL4 protein (RP02179, ABclonal) was dissolved in nuclease-free water and stored at −80°C. Mice were injected intradermally (i.d.) with recombinant ANGPTL4 protein (25 ug/kg/day) or an equal volume of nuclease-free water (control group) every day. Other details were described in Figure 9A. According to the previous description, the Psoriasis Area and Severity Index (PASI) scores of mouse back skin were assessed by two laboratory assistants who did not know the group information (Wang et al., 2018). On the eighth day, plasma samples and the back skin was separated from the sacrificed mice for subsequent detections. Animal experiments were all conducted in accordance with a protocol confirmed by the animal care and use committee of Tongji Medical College of Huazhong University of Science and Technology (Wuhan, China).

### Human Skin Samples

Skin samples were obtained through biopsy and surgical waste from Wuhan Union Hospital. All patients involved signed informed consent. The research protocols were approved by the medical ethics committee of Tongji Medical College of Huazhong University of Science and Technology and the Declaration of Helsinki Principles was followed.

### *In Vitro* Cell Culture and Treatment

HaCaT cell line was purchased from the China Center for Type Culture Collection (Wuhan, China). Cells were incubated in DMEM containing 10% fetal bovine serum (Gibco, CA, United States) and 1% antibiotics (penicillin-streptomycin, Life Technologies) in a humidified incubator with 5% CO_2_ at 37°C. Specific small interfering RNA (siRNA) targeting ANGPTL4 (si-ANGPTL4) was designed and synthesized by Guangzhou RiboBio Co., Ltd. (Guangzhou, China). The si-ANG-01 sequence was GGC​TGG​ACA​GTA​ATT​CAG​A, and si-ANG-02 sequence was CCA​CAA​GCA​CCT​AGA​CCA​T. One batch of HaCaT cells was seeded in 6-well plates and then transfected with 30 nM si-ANGPTL4 or negative control siRNA (si-NC) strictly following the manufacturer’s instructions. The HighGene infection reagent was provided by ABclonal (RM09014). Another batch of HaCaT cells was treated with recombinant ANGPTL4 protein or same volume of PBS. The MEK inhibitor PD0325901 was purchased from Selleck Chemicals (S1036).

### Quantitative Reverse-Transcriptional Polymerase Chain Reaction (qRT-PCR)

Total RNA from skin tissues was extracted with TRIzol reagent (Takara, Kyoto, Japan). Then we used the Prime Script RT reagent Kit with gDNA Eraser (Takara, Kyoto, Japan) to reverse-transcribe RNA into complementary DNA. qRT-PCR was carried out with 2X Universal SYBR Green Fast qPCR Mix (Abclonal, Wuhan, China) and Step-One Real-time PCR systems (Applied Biosystems, CA). The gene expression was analyzed using the 2^−ΔΔCt^ method (Livak and Schmittgen, 2001) for relative quantitation and normalized to Glyceraldehyde 3-phosphate dehydrogenase (GAPDH). The corresponding primers for qRT-PCR used in this study are listed in Supplementary Table S1.

### Western Blotting Analysis

The cultured cells or skin tissues were lysed in RIPA lysis buffer with protease and phosphatase inhibitors. The protein concentration was quantified by bicinchoninic acid (BCA) assay (Boster, California, United States). The protein samples were resolved by 10% SDS-PAGE gels and then transferred to PVDF membranes (Merck Millipore Ltd., Tullagreen, Carrigtwohill, County Cork, Ireland). After blocking in 5% bovine albumin (BSA) for 1 h, membranes were incubated with primary antibodies at against STAT3 (1:1,000, A1192, ABclonal), p-STAT3-Y705 (1:1,000, AP0705, ABclonal), ERK1/ERK2 (1:1,000, A16686, ABclonal), p-ERK1-T202/Y204 + ERK2-T185/Y187 (1:1,000, AP0472, ABclonal), PCNA (1:1,000, A0264, ABclonal), Cyclin D1 (1:1,000, 2922S, Cell Signaling Technology) Cleaved IL-1β (1:1,000, Cell Signaling Technology), IL-17A (1:1,000, A0688, ABclonal), ANGPTL4 (1:1,000, CSB-PA005044, Cusabio), ANGPTL4 (1:500, AF3485, R&D Systems), GAPDH (1:5,000, CSB-MA000071M2m, Cusabio) at 4°C overnight. The membranes were then incubated with HRP-conjugated anti-rabbit/mouse IgG (111-035-003/115-035-003, Jackson ImmunoResearch, West Grove, United States) secondary antibodies for 1 h at room temperature. Images were visualized on Tanon-5200 Chemiluminescent Imaging System (Tanon Science & Technology).

### Histology and Immunohistochemistry

The excised skin of mice was fixed in 10% formalin and embedded in paraffin. For histopathological examination, the tissue slices of 5-μm thickness were stained with hematoxylin and eosin (H&E) under the microscope (Olympus BX61, Tokyo, Japan). Epidermal thickness was calculated based on the means of four random sites of view per tissue and quantitated by ImageJ software as previously described (Liu X. et al., 2017). For immunohistochemistry, the tissue slices of 5-μm thickness were deparaffinized and incubated in hot citric acid buffer (PH 6.0) for antigen retrieval. After immediate cooling, slides were blocked with 5% BSA in TBST for 1 h and incubated with primary antibodies against ANGPTL4 (A2011, ABclonal) and Ki67 (GB13030-2, Servicebio) at 4°C overnight, followed by secondary antibodies at 1:200 for 30 min at room temperature. The immunostaining was visualized by 3,3-diaminobenzidine (Vector Laboratories, Burlington, CA, United States). Quantification of the DAB intensity in the images was performed using ImageJ software.

### Immunofluorescence

The immunofluorescence was performed on skin sections using a primary antibody against ANGPTL4 (1:100, A13425, ABclonal) at 4°C overnight, followed by incubation with a goat anti-rabbit-Cy3 (1:200, GB21303, Servicebio) for 1 h at room temperature. Afterwards, sections were rinsed with PBS three times and then counterstained with 4’,6-diamidino-2-phenylindole fluorescent dye (DAPI). Digital images were taken with confocal laser scanning microscope (Olymbus, Japan) and analyzed by ImageJ software.

### Cell Counting Kit-8 (CCK-8) Assay and Enzyme-Linked Immunosorbent Assay (ELISA)

Cell viability was tested using CCK-8 kit (CK04, Dojindo Laboratories, Kumamoto, Japan). The HaCaT cells were seeded in a 96-well plate at 1 × 10^3^ cells/well. After incubation for 24 h, CCK-8 solution (10 μl) was added to each well. Absorbance value at 450 nm was then detected using a microplate reader (MolecularDevices, Sunnyvale, CA, United States). Plasma ANGPTL4 levels were measured using commercial ELISA kits (MU30102, Bio-Swamp) according to the manufacturer’s instructions. The ELISA kits of IL-1β, IL-17, TNF-α, and IL-6 were purchased from ABclonal (Wuhan, China).

### Statistical Analysis

Data were expressed as the mean ± standard deviation (SD). Statistical analyses were calculated using GraphPad Prism version 9.0 software, San Diego, CA, United States). Comparison was performed with unpaired Student’s *t*-test or one-way analysis of variance (ANOVA). The differences at the 95% confidence level (*p* < 0.05) were considered statistically significant. Pearson’s correlation coefficients for parametric data were calculated to test the relationships between two variables. Simple Linear Regression was employed to detect the association between ANGPTL4 and others factors.

## Results

### Training Set Quality Assessment

The overall flow diagram of this study is presented in Figure 1. In this study, 8 unpaired samples were discarded. Then no sample was removed in GSE30999 by the outliers check (Supplementary Figure S1A). The top 25% of the most variant genes (5,044 genes) were chosen for further WGCNA analysis. Parameter β = 12 (scale-free *R* ^2^ = 0.89) was selected to ensure a scale-free network (Supplementary Figures S1B−E).

### Weighted Co-Expression Network Construction and Identification of Key Modules

**FIGURE 2:**
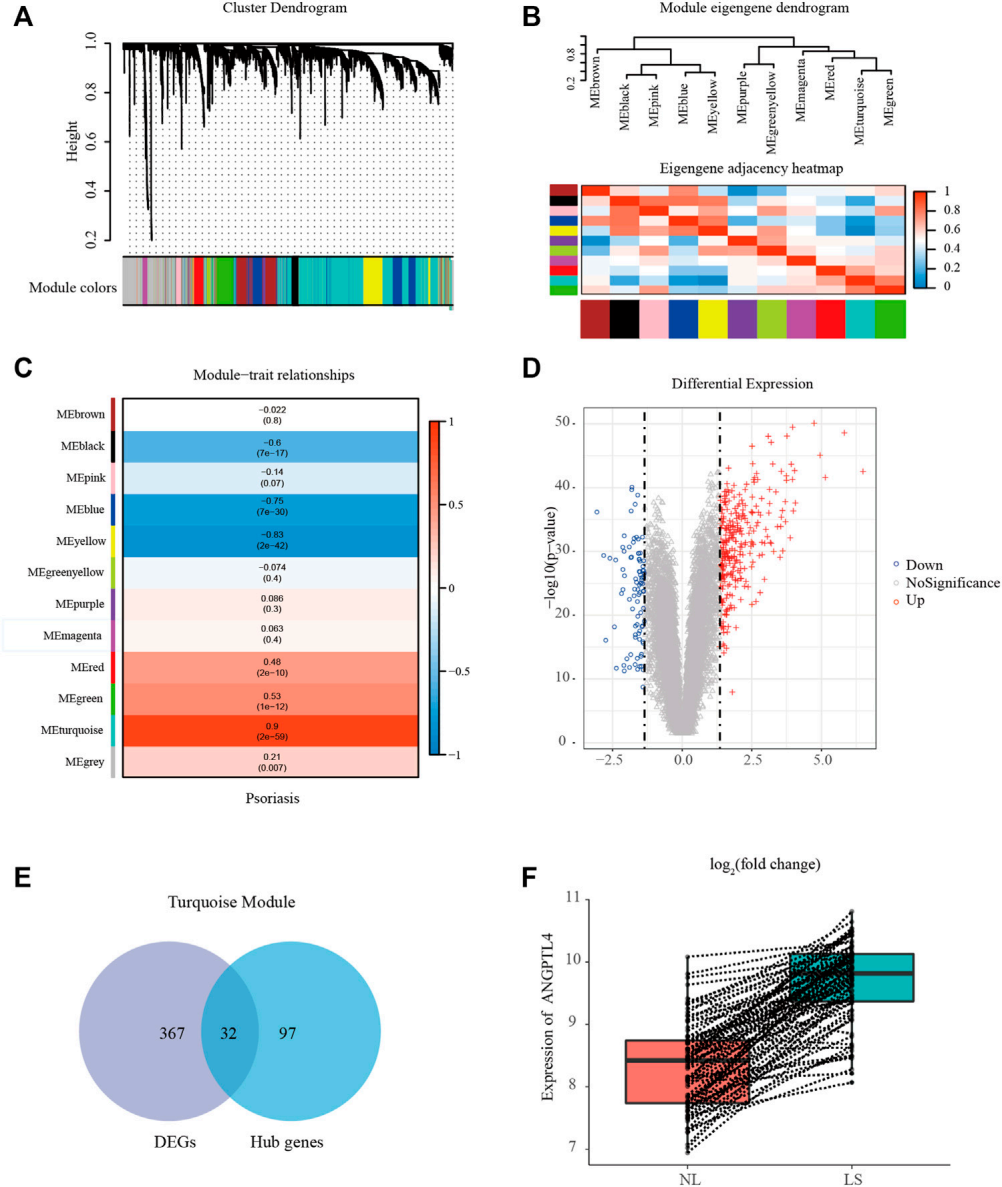
Identification of modules associated with psoriasis using the WGCNA. **(A)** Gene dendrogram of the top 25% most variant genes clustered based on a dissimilarity measure. In total, 12 modules were identified. **(B)** Module eigengene dendrogram and eigengene adjacency heatmap summarize the modules yielded in the hierarchical clustering analysis. **(C)** Heatmap of the module-trait correlation. **(D)** Volcano plot of genes detected in psoriasis. Red means up-regulated DEGs; blue means down-regulated DEGs; grey means no difference. **(E)** Venn diagrams of the intersection between DEGs and the hub genes in the turquoise module. **(F)** Box plot showing the differences in ANGPTL4 gene expression between lesional and paired non-lesional skin in the same patient. LS, psoriasis lesions skin; NL, non-lesional skin; DEG, differentially expressed gene.

We performed WGCNA to classify the genes with similar expression profiles into the same modules by average linkage clustering, and a total of 12 modules were identified (Figure 2A). There were 396 genes in the yellow module, 570 genes in the blue module, 494 genes in the brown module, 311 genes in the green module, 51 genes in the green-yellow module, 786 genes in the grey module, 74 genes in the magenta module, 76 genes in the pink module, 56 genes in the purple module, 156 genes in the red module, 1,963 genes in the turquoise module, and 111 genes in the black module. TOM plots were constructed to present the pairwise gene correlation within each module (Supplementary Figure S2A). Then eigengenes of all modules were calculated, and the dendrogram showed that the 12 modules were divided into two clusters. And the heatmap plotted (Figure 2B) demonstrated similar results. Module eigengene was also calculated for correlation with the clinical trait. Heatmap of module-trait correlation (Figure 2C) showed that the turquoise module was the most highly correlated with the clinical trait. Supplementary Figure S2B demonstrated the gene significance of each module with psoriasis lesions phenotype, and the turquoise module was significant. Only significantly associated genes were considered in this research. Therefore we recognized the turquoise module for further analysis.

### Hub Gene Identification and Functional Enrichment Analysis

In the turquoise module, a total of 129 genes among the top 3 sub-networks were identified as hub genes by “MCODE” plugins in Cytoscape. The representative genes were shown in Supplementary Figure S2C. A total of 399 DEGs (310 up-regulated and 89 down-regulated) were chosen for subsequent analysis (threshold adjusted *p*-value ≤ 0.05, |log_2_ FC|≥1.35). Figure 2D showed the volcano plot of all DEGs. Eventually, 32 common genes between the hub genes in the turquoise module and DEGs were screened as the final candidates (Figure 2E). More detailed gene information was available in Supplementary Table S2. These overlapped candidate hub genes were picked and their literature content was analyzed. ANGPTL4 regulates inflammation, proliferation, lipid metabolism, vessel permeability, and tumor progression. Our previous study has found ANGPTL4 as a key regulator in diabetic cardiomyopathy *via* FAK/SIRT3/ROS pathway in cardiomyocyte (Dai et al., 2021). ANGPTL4 also stimulates STAT3-mediated iNOS expression and enhances angiogenesis to accelerate wound healing in diabetic mice (Chong et al., 2014). What’s more, ANGPTL4 could interact with integrins β1 and β5 to modulate keratinocyte migration (Goh et al., 2010a). Therefore, we selected ANGPTL4 to explore its possible role in psoriasis. Figure 2F showed the differences in ANGPTL4 gene expression between lesional and paired non-lesional skin in the same patient.

**FIGURE 3:**
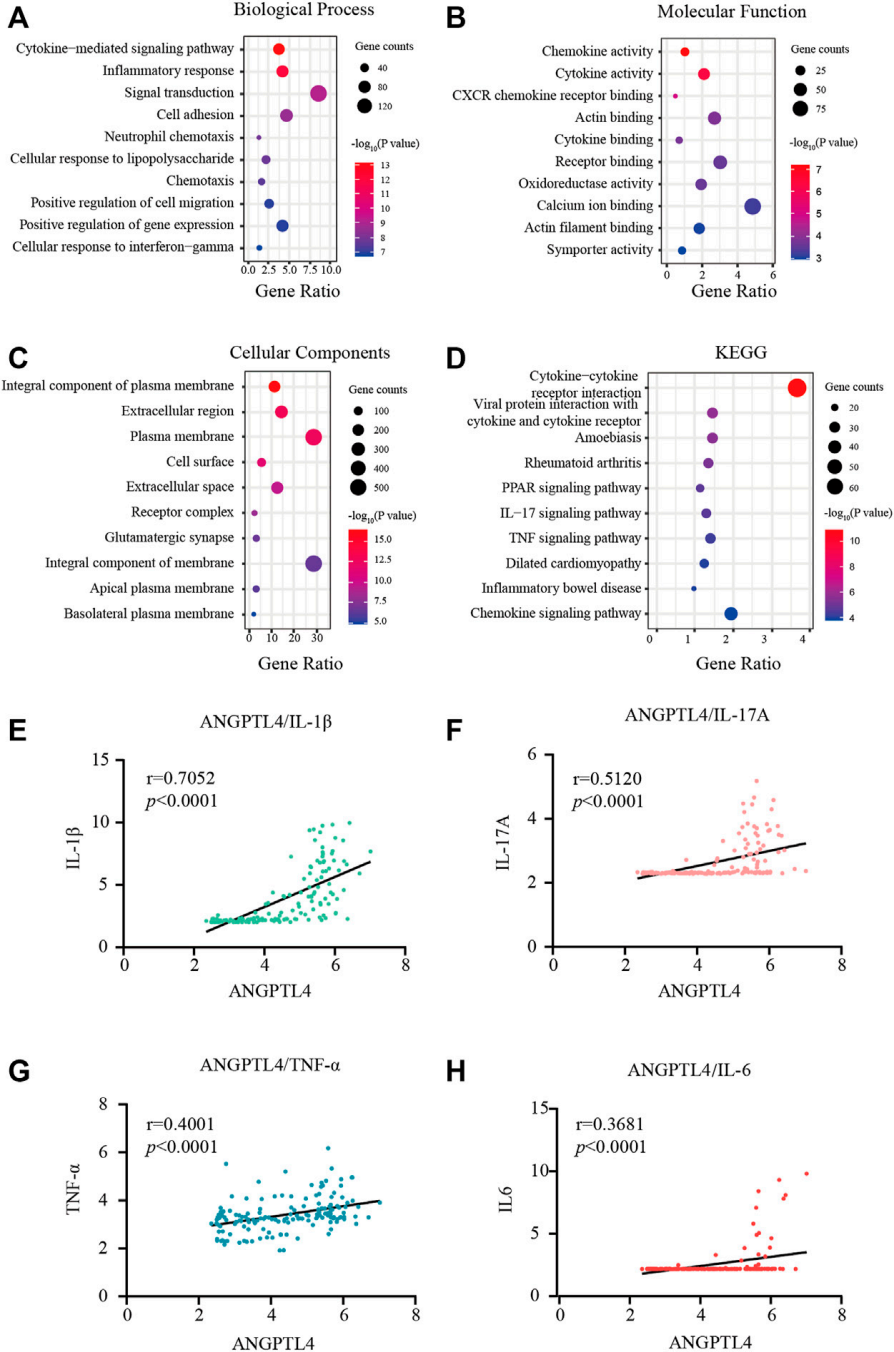
Functional enrichment analysis. **(A–C)** GO terms of Biological process, Cellular component, and Molecular function for genes in the turquoise module. **(D)** KEGG analysis for genes in the turquoise module. **(E–H)** Correlation analysis between the levels of ANGPTL-4 and IL-1β, ANGPTL-4 and IL-17A, ANGPTL-4 and IL-6, ANGPTL-4 and TNF-α in the skin tissues. KEGG, Kyoto Encyclopedia of Genes and Genomes; GO, Gene Ontology.

Figures 3A–D showed the most significant GO term and KEGG pathways of all genes in the turquoise module. Among biological processes, cytokine-mediated signaling pathway and inflammatory response were significantly associated with the cluster (Figure 3A). In molecular function analysis, the results showed that the genes were significantly associated with chemokine activity and cytokine activity (Figure 3B). Among cellular component enrichment analysis, the integral component of plasma membrane and extracellular region were enriched (Figure 3C). As to KEGG pathway analysis, cytokine-cytokine receptor interaction was mainly associated with these genes (Figure 3D). Psoriasis is an autoimmune inflammatory disease characterized by cytokines elevated, including TNF-α, IL-1β, IL-6, and IL-17A (Arican et al., 2005). To elucidate the relationships between the levels of inflammatory cytokines and ANGPTL-4 in the skin tissues. We peformed the correlation analysis. The results showed the levels of ANGPTL-4 have a positive correlation with IL-1β (*p* < 0.0001), IL-17A (*p* < 0.0001), TNF-α (*p* < 0.0001) and IL-6 (*p* < 0.0001) in the skin tissues (Figures 3E–H).

### Increased Expression of ANGPTL4 in the Lesion Skin of Imiquimod-Treated Mice and Psoriasis Patients

**FIGURE 4:**
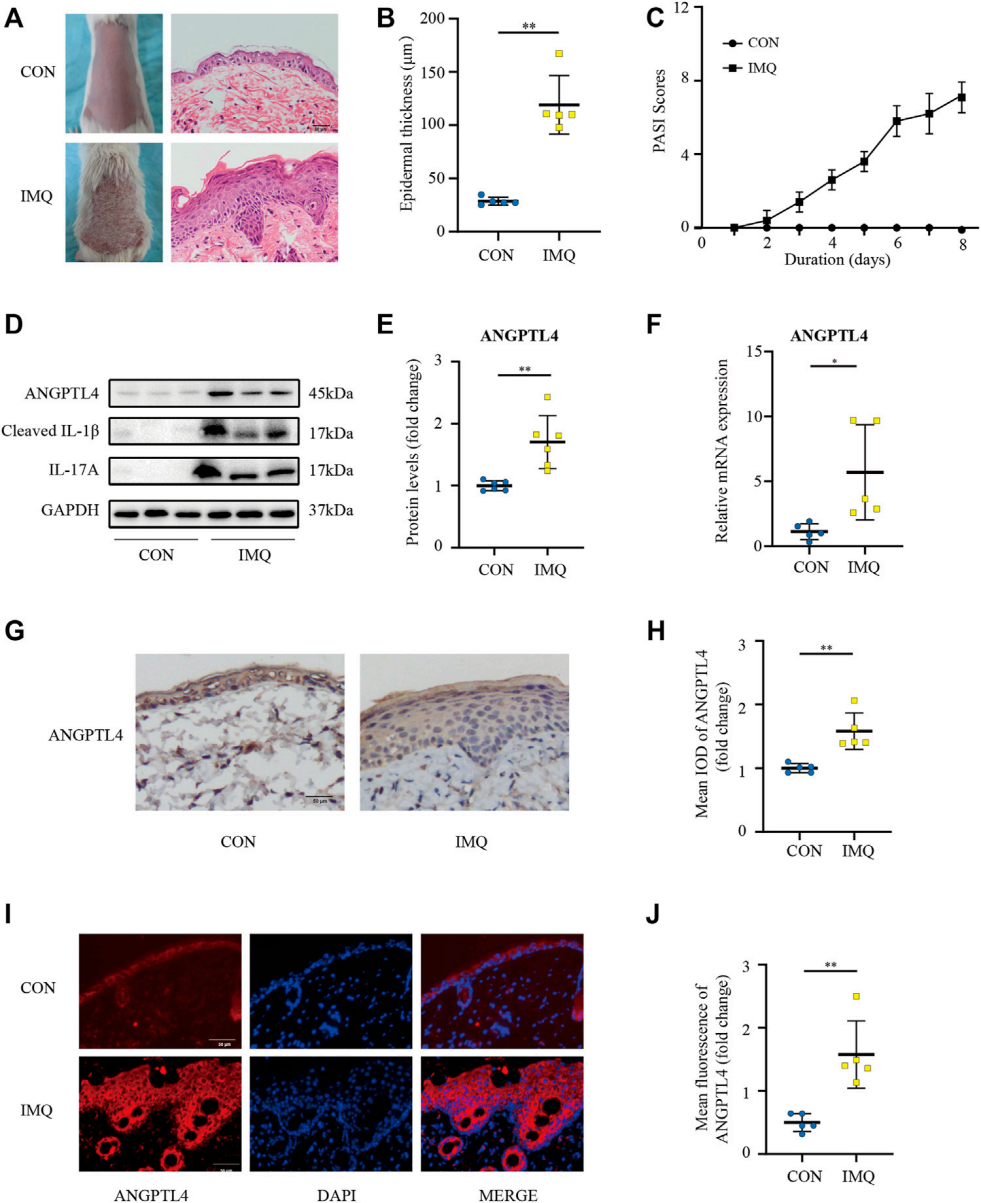
ANGPTL4 expression is elevated in IMQ-induced psoriasiform dermatitis in mice. IMQ or control cream (vaseline) was applied daily to the dorsal skin for female BALB/c mice. **(A)** Phenotypical presentation of mouse back skin and light microscopy examination of skin sections stained with H&E after 7 days of control cream (upper panel) or IMQ treatment (lower panel). Scale bars, 50 μm. **(B)** The skin thickness was measured on day 8. Significant differences are indicated (*n* = 5). **(C)** Daily assessment of epidermal erythema, scales, and thickeness of the shaved backs. PASI score was calculated to assess the severity of psoriasis by adding the scores of three criteria (range from 0 to 12). **(D,E)** Representative immunoblotting and analysis of ANGPTL4 from skin samples of control cream or IMQ treated mice on day 8 (*n* = 6). **(F)** Relative mRNA expression of ANGPTL4 in IMQ treatment mice and control cream on day 8 (*n* = 5). **(G,H)** Representative immunohistochemical stainings and quantification of ANGPTL4 in skin sections (*n* = 5). Scale bars, 50 μm. **(I,J)** Representative images and quantification of ANGPTL4 fluorescence in skin sections. The nuclei were detected by DAPI (blue). Scale bar, 50 μm. All data were shown as mean ± standard deviation (SD). IMQ, imiquimod; H&E, haematoxylin and eosin; IOD, the integral optical density. *p < 0.05, **p < 0.01 vs. CON group.

**FIGURE 5:**
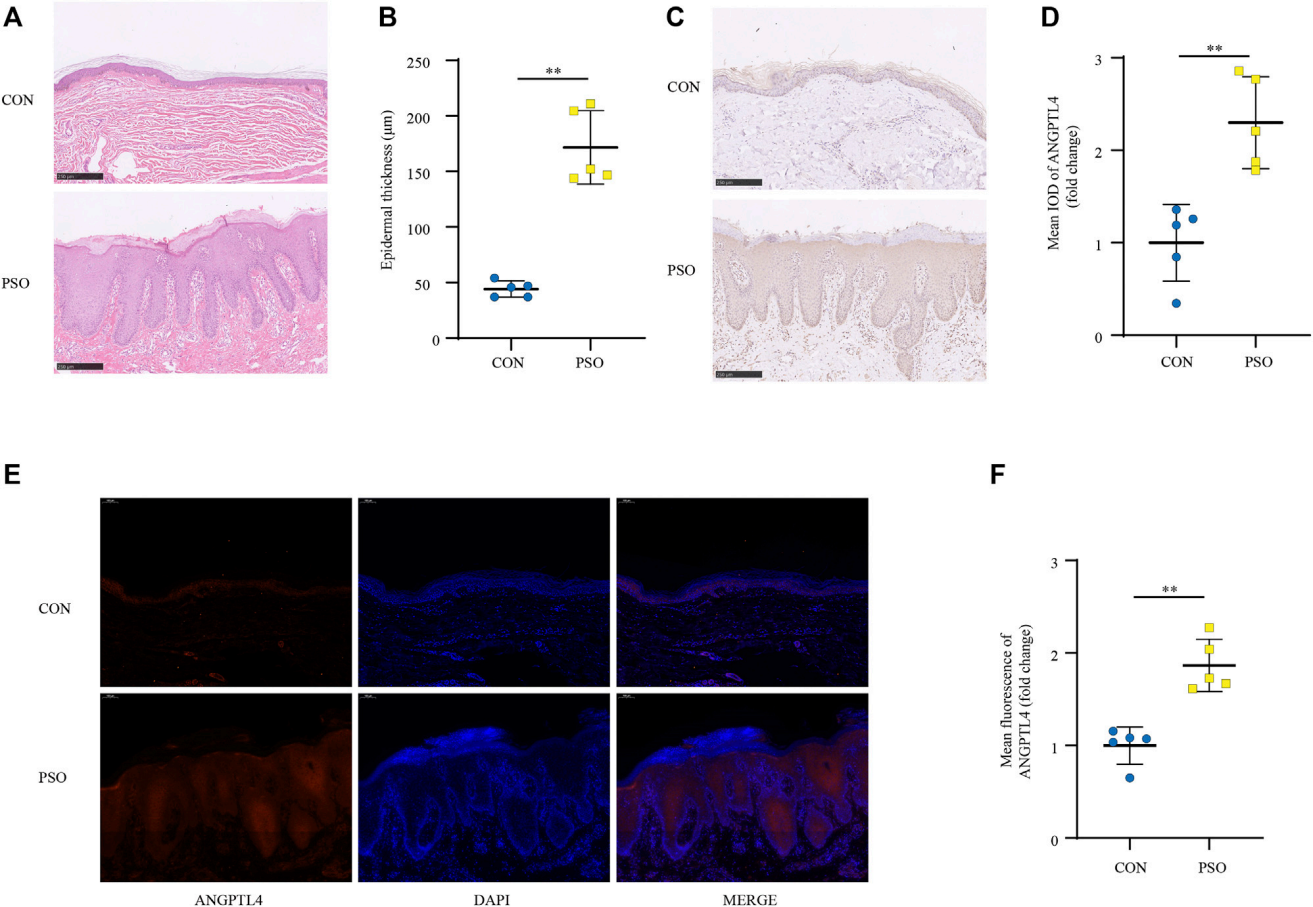
ANGPTL4 expression is elevated in psoriasis patients. **(A)** Phenotypical presentation of psoriasis and normal skin stained with H&E. Scale bars, 250 μm. **(B)** The skin thickness was measured. Significant differences are indicated (*n* = 5). **(C,D)** Representative immunohistochemical stainings and quantification of ANGPTL4 in skin sections from psoriasis and non-psoriasis patients (*n* = 5). Scale bars, 250 μm. **(E,F)** Representative images and quantification of ANGPTL4 fluorescence in skin sections. The nuclei were detected by DAPI (blue). Scale bar, 100 μm. All data were shown as mean ± standard deviation (SD). H&E, haematoxylin and eosin; IOD, the integral optical density. **p < 0.01 vs. CON group.

IMQ-induced skin inflammation is a well-characterized mouse model for psoriasiform dermatitis (van der Fits et al., 2009). To examine the ANGPTL4 expression profile *in vivo*, we established psoriasiform dermatitis animal models by inducing the skin of mice with IMQ. The backs of mice were topically treated with IMQ/Vaseline for seven consecutive days and sacrificed to harvest skin samples on day 8. As shown in Figures 4A,C, we found that IMQ-treated skin developed signs of erythema, scaling, and thickening over the course of IMQ treatment, while control (CON) mice did not show any signs of skin lesion. Compared with healthy skin, the psoriasiform skin showed apparent epidermal thickening (Figure 4B). The western blot and qRT-PCR results showed an obviously increased expression of ANGPTL4 and inflammatory cytokines within the psoriasis-like skin lesions, demonstrating that ANGPTL4 was stimulated in a psoriasis-like inflammatory environment (Figures 4D–F). In addition, we examined the mice’s plasma ANGPTL4 levels. The ELISA result showed significantly higher levels of ANGPTL4 in the plasma after IMQ treatment. Western blot results of plasma ANGPTL4 also demonstrated similar results (Supplementary Figures 3A–C). The immunohistochemistry and immunofluorescence results revealed a corresponding elevation of ANGPTL4 expression, and the expression was significantly increased in the epidermis of psoriasiform lesions than that in normal skin (Figures 4G–J). To clarify the expression and localization of ANGPTL4 in psoriasis, we then collected skin samples from psoriasis and non-psoriasis patients for immunohistochemistry and immunofluorescence staining. The skin of the psoriasis patients showed obviously thickened epidermis (Figures 5A,B). The results showed that the staining intensity of ANGPTL4 in the epidermis of psoriatic lesions was greater than that of normal skin (Figures 5C–F). ANGPTL4 was dominantly expressed in the cytoplasm of the epithelial cells (Figure 5E). Together, these results verified that ANGPTL4 was upregulated in psoriasis, suggesting a potential role of ANGPTL4 in psoriasis pathogenesis.

### ANGPTL4 Promoted the Proliferation and Affected the Expression of Inflammatory Cytokines in Human Keratinocyte Cells

**FIGURE 6:**
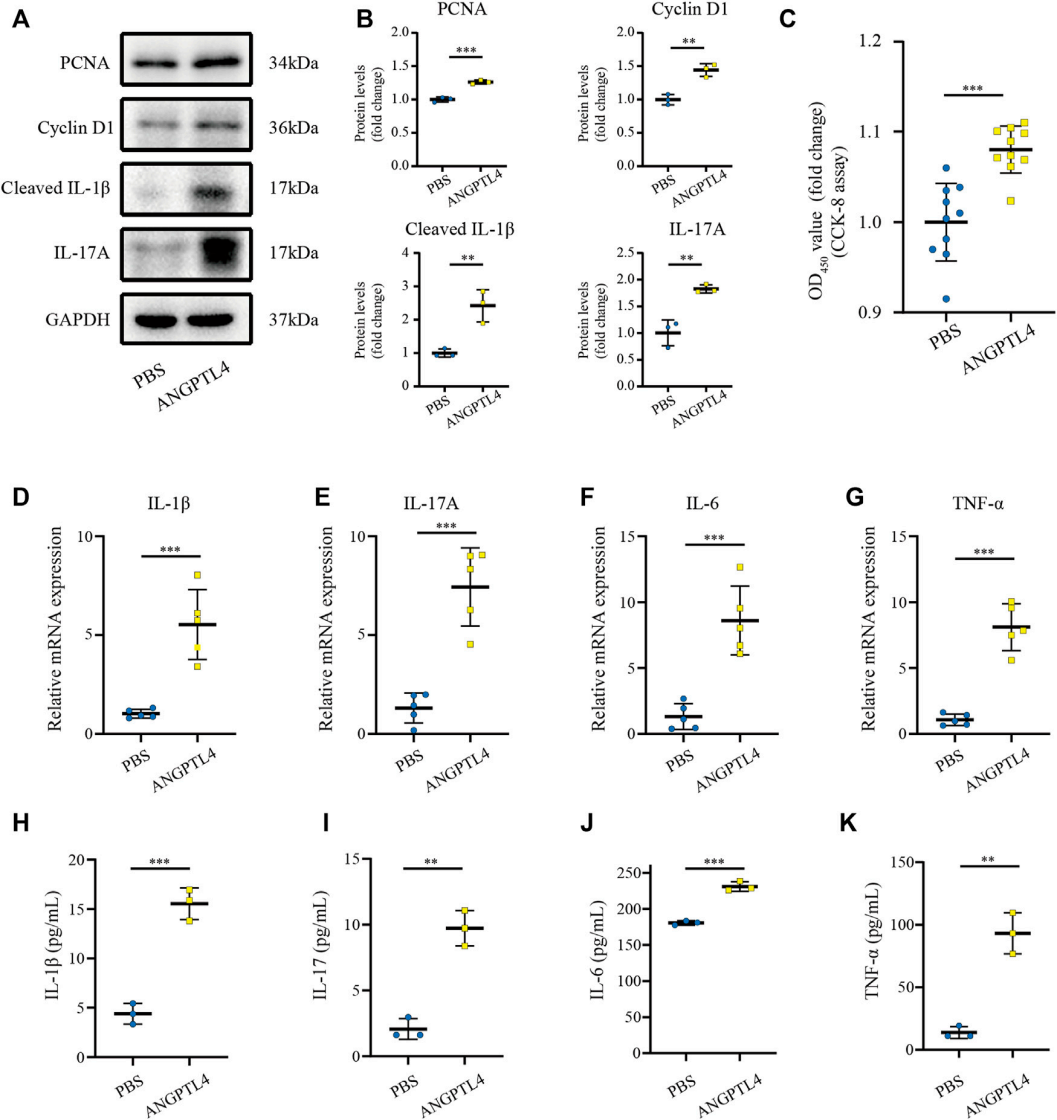
Effect of ANGPTL4 on Human Keratinocyte Cells **(A)** Representative Western blots showing PCNA, Cyclin D1, Cleaved IL-1β and IL-17A in HaCaT cells after treatment with ANGPTL4 (500 ng/ml). **(B)** WB analysis of PCNA, Cyclin D1, Cleaved IL-1β, IL-17A expression, respectively. **(C)** CCK-8 assay results (*n* = 10). **(D–G)** Relative mRNA expression of IL-1β, IL-17A, IL-6 and TNF-α normalized to a GAPDH internal control (*n* = 5). Values are expressed relative to control groups. **(H–K)** The secretion of IL-1β, IL-17, IL-6 and TNF-α were determined by ELISA (*n* = 3). Results shown are representative data of three independent experiments. All data were shown as mean ± standard deviation (SD). **p < 0.01, ***p < 0.001 vs. CON group.

**FIGURE 7:**
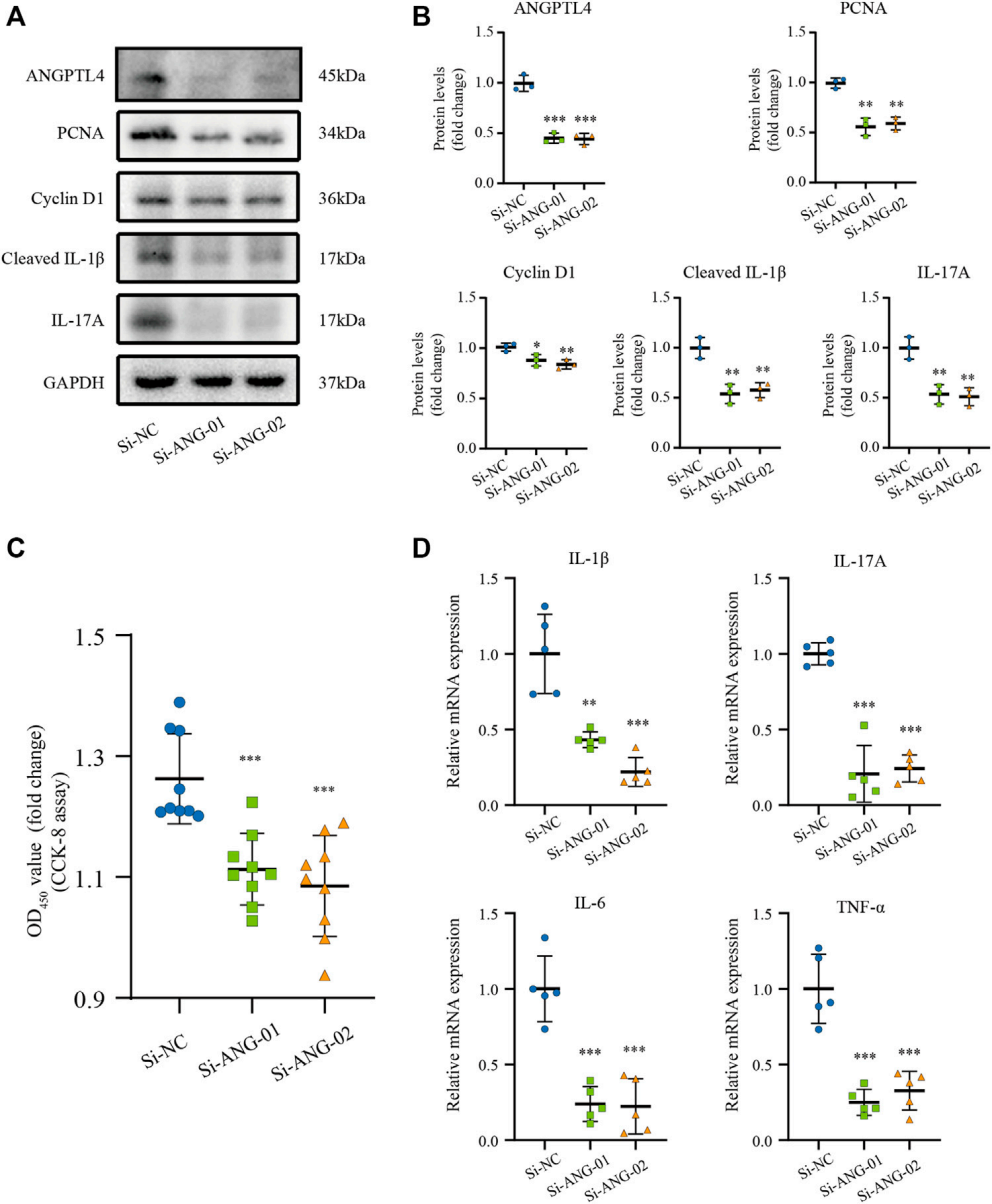
Effect of ANGPTL4 Knockdown on Human Keratinocyte Cells **(A,B)** Representative Western blots and quantification of ANGPTL4, PCNA, Cyclin D1, Cleaved IL-1β and IL-17A in cultured HaCaT cells under the treatment with si-RNA targeting ANGPTL4 (si-ANG-01 and si-ANG-02) or negative control siRNA (si-NC). GAPDH was used as loading control (*n* = 3). **(C)** CCK-8 assay results (*n* = 9). **(D)** Relative mRNA expression of IL-1β, IL-17A, IL-6 and TNF-α normalized to a GAPDH internal control (*n* = 5). Results shown are representative data of three independent experiments. All data were shown as mean ± standard deviation (SD). *p < 0.05, **p < 0.01, ***p < 0.001 vs. CON group.

Previous studies have indicated that ANGPTL4 is involved in the regulation of inflammation and proliferation (Pal et al., 2011; Cho et al., 2019). By the bioinformatic analysis, we found the function of the turquoise module focuses on inflammatory response. Therefore, we hypothesize that ANGPTL4 could participate in psoriasis *via* regulating the keratinocytes proliferation and the expression of inflammatory cytokines in the skin. In order to prove our hypothesis, *in vitro* cell culture experiments were further applied. As ANGPTL4 is a secreted protein, we decided to use recombinant ANGPTL4 protein to evaluate its function. Indeed, western blot analysis showed that the ANGPTL4-treated group had upregulated proliferation-related proteins (PCNA, Cyclin D1) and increased levels of cleaved IL-1β and IL-17A in the HaCaT cells compared with the control group (Figures 6A,B). Consistent with the above findings, CCK-8 assay result showed ANGPTL4 treatment increased proliferative capacity of HaCaT cells (Figure 6C) and qRT-PCR showed that the mRNA expression levels of IL-1β, IL-17A, TNF-α, and IL-6 were significantly increased in ANGPTL4-treated HaCaT cells in contrast to the normal controls (Figures 6D–G). What’s more, ANGPTL4 increased the secretion of pro-inflammatory cytokines in the cell culture supernatants, as revealed by ELISA (Figures 6H–K). Transfection with two independent siRNA for ANGPTL4, namely, si-ANG-01 and si-ANG-02, significantly decreased ANGPTL4 protein expression compared with the control siRNA group (*p* < 0.001) (Figures 7A,B). Western blot was used to determine the expression of proliferation markers (PCNA, Cyclin D1) and inflammation-related proteins (cleaved IL-1β, IL-17A) after transfection with si-ANG-01 and si-ANG-02 (Figures 7A,B). As expected, silencing of ANGPTL4 effectively inhibited target gene protein expression of proliferation and inflammation in human keratinocyte cells. CCK-8 assay result showed silencing of ANGPTL4 could inhibit proliferative capacity of HaCaT cells effectively (Figure 7C) and the mRNA expression levels of IL-1β, IL-17A, TNF-α, and IL-6 were significantly decreased (Figure 7D).

### ANGPTL4 Modulated the Proliferation and Inflammation *via* ERK1/2 and STAT3 Pathways in Human Keratinocyte Cells *In Vitro*

**FIGURE 8:**
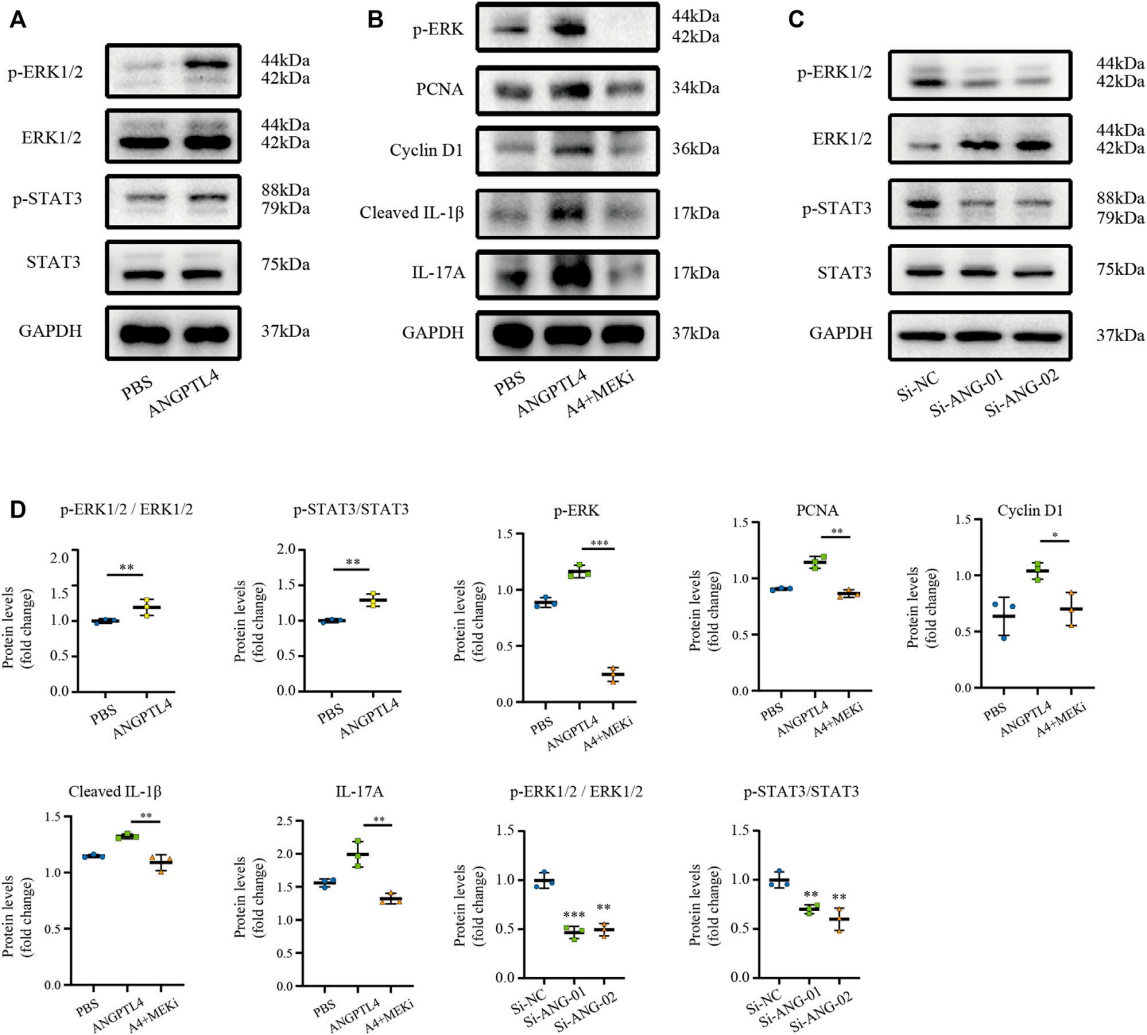
Modulation of STAT3 and ERK1/2 pathways by ANGPTL4 in HaCaT cells. **(A)** Representative Western blots showing p-ERK1/2, ERK1/2, p-STAT3, STAT3 levels in HaCaT cells after treatment with ANGPTL4 (500 ng/ml). **(B)** Representative Western blots showing PCNA, Cyclin D1, Cleaved IL-1β and IL-17A in HaCaT cells after treatment with ANGPTL4 and MEK inhibitor (PD0325901). **(C)** Representative Western blots showing p-ERK1/2, ERK1/2, p-STAT3 and STAT3 levels in HaCaT cells under treatment with si-RNA targeting ANGPTL4 (si-ANG-01 and si-ANG-02) or negative control siRNA (si-NC). GAPDH was used as loading control (*n* = 3). **(D)** Quantitative Western blot analysis. Results shown are representative data of three independent experiments. All data were shown as mean ± standard deviation (SD). *p < 0.05, **p < 0.01, ***p < 0.001 vs. CON group.

Extracellular regulated protein kinase 1/2 (ERK1/2) pathway and signal transducer and activator of transcription 3 (STAT3) signaling have been implicated in ANGPTL4-mediated physiological and pathological processes (Chong et al., 2014; Zhu et al., 2016; Zhang et al., 2021). Therefore, we selected ERK1/2 and STAT3 pathways as candidate pathways involved in ANGPTL4-mediated regulation of proliferation and inflammation. Western blot assay showed that ANGPTL4 treatment induced phosphorylation of ERK1/2 and STAT3 in human keratinocyte cells *in vitro*. And inhibition of MEK with PD0325901 could reverse the effects of ANGPTL4 in proliferation and inflammation (Figures 8A,B). As demonstrated in Figure 8C, phosphorylation of ERK1/2 and STAT3 were inhibited after transfection with si-ANG-01 and si-ANG-02. Protein levels were statistically quantified (Figure 8D). These results suggested that ANGPTL4 may promote proliferation and inflammation *via* ERK1/2 and STAT3 signaling pathways in psoriasis.

### Role of ANGPTL4 in IMQ-Induced Psoriasiform Dermatitis in Mice

**FIGURE 9:**
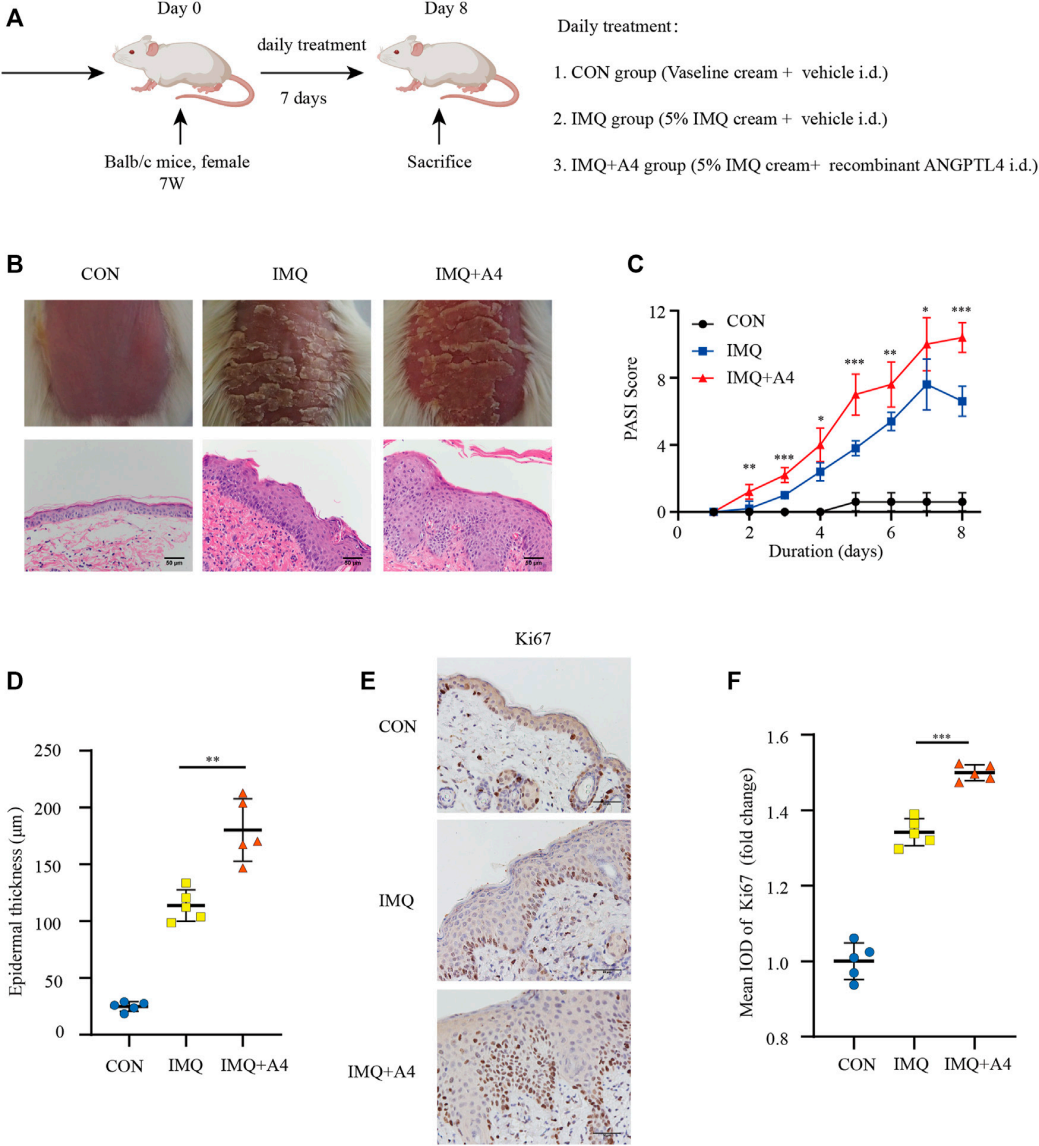
Role of ANGPTL4 in the IMQ-induced psoriasiform dermatitis in mice. **(A)** Schematic illustration of the animal experiment protocol for the CON, IMQ, IMQ + A4 group (*n* = 5). **(B)** Phenotypical presentation of mouse back skin and light microscopy examination of skin sections stained with H&E after 7 days of CON, IMQ, IMQ + A4 groups. Scale bars, 50 μm. **(C)** Daily assessment of epidermal erythema, scales, and thickness of the shaved backs. PASI score was calculated to assess the severity of psoriasis by adding the scores of three criteria (range from 0 to 12). **(D)** The skin thickness was measured on day 8. Significant differences are indicated. **(E,F)** Representative immunohistochemical stainings and quantification of ANGPTL4 in skin sections. Scale bars, 50 μm. A4, ANGPTL4; CON group = 62.5 mg/day Vaseline cream +25 ug/kg/day vehicle i. d. for 7 days, IMQ group = 62.5 mg/day 5% IMQ cream +25ug/kg/day vehicle i. d., IMQ + A4 group = 62.5 mg 5% IMQ cream+ 25 ug/kg/day recombinant ANGPTL4 i. d. Data were shown as mean ± standard deviation (SD); *p < 0.05, **p < 0.01, ***p < 0.001 vs. CON group.

Psoriasiform dermatitis models in mice were induced by IMQ treatment to investigate the effect of ANGPTL4 on psoriasis *in vivo* (Figure 9A). Compared with the IMQ group, the intradermal injection with recombinant ANGPTL4 protein significantly exacerbated the clinical phenotype of IMQ-induced psoriasiform dermatitis, which included redness, thickening, and scaling of the skin (Figure 9B). Histological results showed recombinant ANGPTL4 protein significantly aggravated the histologic changes caused by IMQ compared with the IMQ group. It showed much parakeratosis and much inflammatory cell infiltration than the IMQ group. Similarly, PASI scores were higher in the IMQ + ANGPTL4 group than in the IMQ group (Figure 9C), while the IMQ + ANGPTL4 group showed much epidermal thickening compared with the IMQ group (Figure 9D).

**FIGURE 10:**
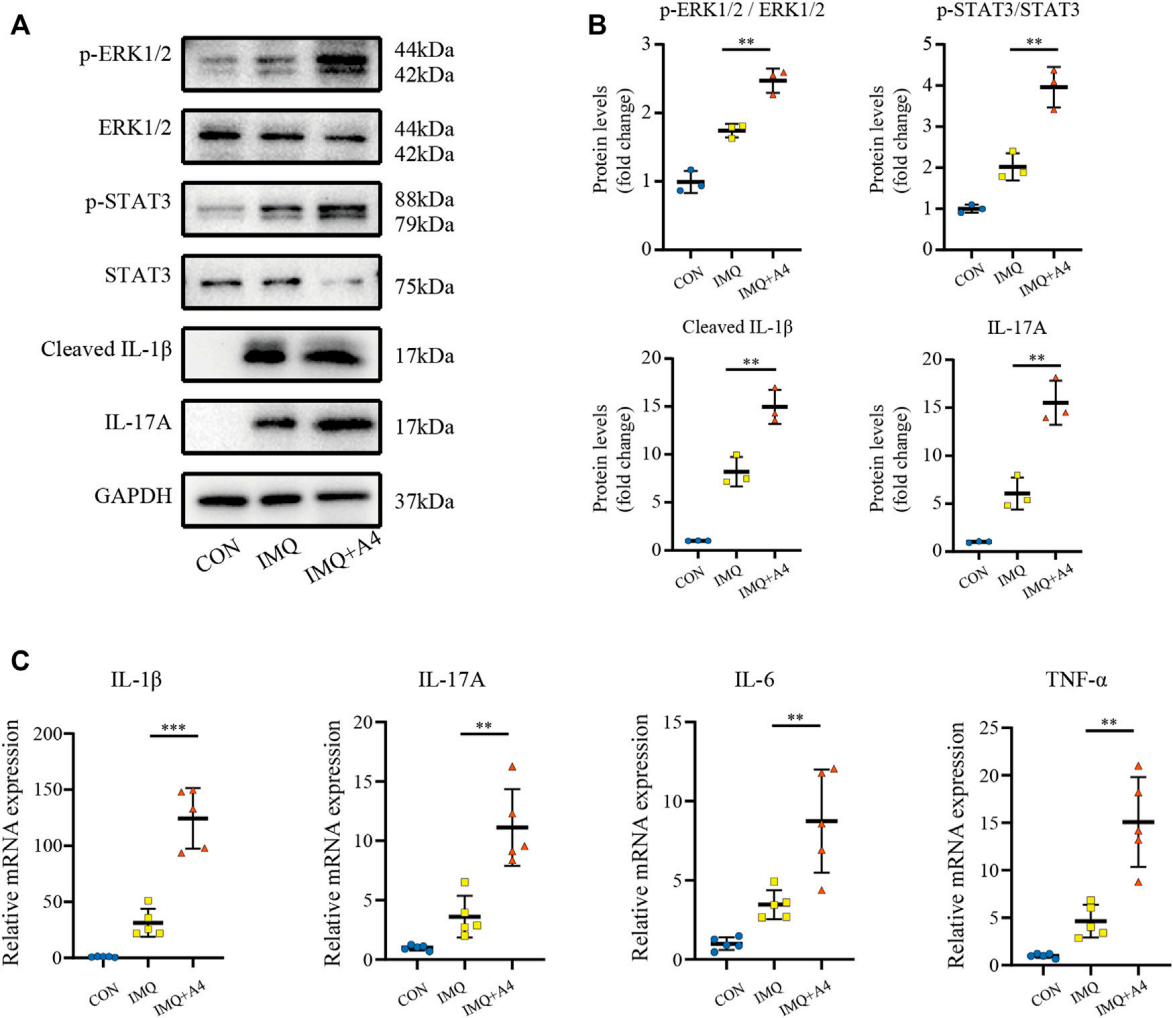
Role of ANGPTL4 in the IMQ-induced psoriasiform dermatitis in mice. **(A,B)** Representative Western blots and quantification showing phospho-ERK1/2, ERK1/2, phospho-STAT3, STAT3, Cleaved IL-1β and IL-17A levels in the CON, IMQ, IMQ + A4 group (*n* = 3). **(C)** Relative mRNA expression of IL-1β, IL-17A, IL-6 and TNF-α in the CON, IMQ, IMQ + A4 group (*n* = 5). Data were shown as mean ± standard deviation (SD); **p < 0.01, ***p < 0.001 vs. CON group.

To evaluate proliferation after intradermally injecting recombinant ANGPTL4 protein immunohistochemical staining for the proliferative marker Ki67 was performed. As illustrated in Figures 9E,F, the expression of Ki-67 was increased in recombinant ANGPTL4-injected mice. Finally, the protein levels of p-ERK1/2, ERK1/2, p-STAT3, STAT3, cleaved IL-1β, and IL-17A were assessed by western blot (Figures 10A,B), and the mRNA expression levels of IL-1β, IL-17A, TNF-α, and IL-6 were detected by qRT-PCR (Figure 10C). Consistent with our *in vitro* findings, the protein and mRNA levels of pro-inflammatory cytokines were significantly upregulated, as well as the p-ERK1/2/ERK1/2 ratio and p-STAT3/STAT3 ratio, suggesting that ANGPTL4 promoted the proliferation and affected the expression of inflammatory cytokines by ERK1/2 and STAT3 signaling pathways *in vivo*. These results indicated that ANGPTL4 modulated the clinical severity of IMQ-induced psoriasiform dermatitis in mice.

## Discussion

In our study, we constructed WGCNA analysis based on the GSE30999 dataset. We have identified 5,044 differential genes between the psoriasis lesions and the non-lesional skin samples. The turquoise module was selected as the key module mainly involved in psoriasis. We then applied GO and KEGG analysis to elucidate the biological functions of genes in the turquoise module. The results indicated that the turquoise module was mainly related to inflammatory response. Among these overlapped candidate hub genes in the turquoise module, we eventually selected ANGPTL4 to explore its possible role in psoriasis. The present study found that ANGPTL4 was significantly upregulated in psoriasis patients and IMQ-induced psoriasiform dermatitis in mice. Furthermore, ANGPTL4 could promote keratinocyte proliferation and induce inflammatory response *via* ERK1/2 and STAT3 dependent signaling pathway, resulting in pathological changes in psoriasis. To the best of our knowledge, this is the first study showing that ANGPTL4 plays a major pathological role in psoriasis.

ANGPTL4 belongs to the angiopoietin-like protein family. Angiopoietin-like proteins (ANGPTL1-8) share high similarities with angiopoietin proteins but do not bind to angiopoietin receptors Tie1 or Tie2 (Grootaert et al., 2012; Zhu et al., 2012). ANGPTL4 is a multifaceted secreted protein that regulates lipid metabolism, vessel permeability, inflammation, proliferation, and tumor progression (La Paglia et al., 2017; Aryal et al., 2019). Previous studies have shown that the expression of ANGPTL4, relatively rare in normal intact skin tissues, was remarkably increased during the re-epithelialization phase of wound healing (Goh et al., 2010b). Similarly to this, our results revealed that ANGPTL4 expression was markedly elevated both in skin and plasma in IMQ-induced psoriasiform mice.

Psoriasis is characterized by aberrant inflammation and epidermal hyperplasia (Peinemann et al., 2021). It was reported that the largest alteration in cell cycle was shortened from 311 h in basal keratinocytes of normal lesions to 36 h in psoriatic lesions (Weinstein et al., 1985; Ogawa et al., 2018), indicating substantial acceleration in cell proliferation in psoriatic lesions. The acceleration of keratinocyte proliferation is considered to involve the pathomechanism underlying psoriasis. Early studies have provided evidence for the involvement of ANGPTL4 in cellular proliferation and differentiation during wound healing process (Goh et al., 2010a; Goh et al., 2010b; Pal et al., 2011). It is still unclear whether ANGPTL4 contributes to the proliferation of epidermis in psoriasis. Immunohistochemistry and immunofluorescence results in the present study showed that ANGPTL4 was mainly expressed in keratinocytes of psoriasiform skin. In addition, *in vitro* experiments using recombinant ANGPTL4 protein revealed that ANGPTL4 markedly promoted the proliferation of human keratinocyte cells while silencing of ANGPTL4 effectively attenuated target gene protein expression of proliferation.

Apart from abnormal proliferation, epidermal keratinocytes also play an essential role in initiating and amplifying inflammation (Xu et al., 2018). Studies have reported that ANGPTL4 can exert pro-inflammatory effects on various experimental animal models of inflammation. Qin and colleagues found knockdown of ANGPTL4 inhibited high glucose-induced cell proliferation and inflammatory response in glomerular mesangial cells (Qin et al., 2019). And Jung et al. (2020) demonstrated that ANGPTL4 induced pancreatitis and accelerated the pathological process of acute pancreatitis severity by inducing acinar cell injury and releasing massive inflammatory cytokines. These aforementioned results are consistent with the pro-inflammatory effect of ANGPTL4 in our studies. We demonstrated that recombinant ANGPTL4 protein could significantly promote the expression of inflammatory cytokines of keratinocytes. Furthermore, a significant positive correlation between the levels of IL-1β, IL-17A, TNF-α, IL-6 and ANGPTL4 found in our bioinformatic analysis also supports the conclusion. It suggests that ANGPTL4 might be related to inflammation in the pathophysiological mechanism of psoriasis. All of these results drive us to believe that keratinocytes ANGPTL4 may play a significant role in psoriasis development.

Cardiovascular disease is a co-morbidity in psoriasis, and ANGPTL4 has been strongly linked with the development of atherosclerosis and type 2 diabetes. In our study, we found ANGPTL4 elevated in the skin and plasma of psoriasiform dermatitis models. The elevated expression of ANGPTL4 in plasma may lead to decreased plasma lipoprotein lipase (LPL) activity, decreased triacylglycerol (TAG) clearance, and increased plasma TAG levels (Aryal et al., 2019). Human genetics studies show that inactivating mutations in the ANGPTL4 gene (E40K) improve glucose and lipid homeostasis and reduce the risk of coronary artery disease and diabetes (Dewey et al., 2016; Stitziel et al., 2016; Liu D. J. et al., 2017; Gusarova et al., 2018). Therefore, ANGPTL4 may also be an important bridge between psoriasis and cardiovascular disease.

Nevertheless, inconsistent reports exist in the literature. Cho et al. (2019) have demonstrated ANGPTL4 secreted from mesenchymal stem cells has a potentially anti-inflammatory role in a pathological microenvironment, and ANGPTL4 could regulate inflammation-related gene expression in macrophages. The distinct functions may be strongly dependent on the pathological conditions, which could be due to different isoforms of ANGPTL4 in complex processes, such as protein modifications (Yang et al., 2008) and post-translational cleavage of the protein (Jung et al., 2020).

Though several studies have explored the mechanism of ANGPTL4 in various disease models, the underlying mechanism in psoriasis remains elusive. In this study, the ERK and STAT3 signaling were found to be activated by ANGPTL4 stimulation *in vitro* and *in vivo.* Consistently, the induction of ANGPTL4 expression was reported dependent on the activation of ERK signaling in different cell types (Stapleton et al., 2010; Katanasaka et al., 2013; Noh et al., 2015). And ERK pathway has been implicated in ANGPTL4-mediated colorectal cancer metastasis (Zhang et al., 2021), angiogenesis (Yang et al., 2008) as well as cell proliferation (Zhu et al., 2016). On the other hand, substantial evidence has shown that STAT3 is crucial in the development and pathogenesis of psoriasis (Calautti et al., 2018). STAT3 also has been shown to be involved in ANGPTL4-mediated wound healing (Chong et al., 2014). Taken together, ANGPTL4 could modulate the proliferation and inflammation by ERK1/2 and STAT3 signaling pathways.

These data offer the first hint that ANGPTL4 could be a critical player in psoriatic inflammation and epithelial proliferation. However, several limitations of this study should be acknowledged. First, the sizes of human biological samples were not large enough. Second, there are no known specific inhibitors of ANGPTL4. Though we have performed RNA interference knockdown of ANGPTL4 in HaCaT cells *in vitro*, knockdown of ANGPTL4 in IMQ-treated mice should be used to better validate the function of the ANGPTL4 in the future. Further *in vivo* experiments and studies are needed to confirm the clinical value of ANGPTL4 in psoriasis.

In conclusion, we demonstrated that ANGPTL4 participates in the pathogenesis of psoriasis. ANGPTL4 expression was significantly elevated in both plasma and IMQ-induced psoriasiform skin of mice. ANGPTL4 could promote keratinocyte proliferation and significantly upregulate the levels of pro-inflammatory factors *in vitro* and *in vivo*. Mechanistically, ANGPTL4 modulated the proliferation and inflammation by ERK1/2 and STAT3 signaling pathways*.* Our study suggests that ANGPTL4 may be a promising therapeutic target for psoriasis in the future.

## Data Availability

Publicly available datasets were analyzed in this study. This data can be found here: GEO, GSE30999. The original contributions presented in the study are included in the article/Supplementary Materials, further inquiries can be directed to the corresponding authors.

## Supplementary Material

Supplementary Figure S1
**(A)** Samples clustering to detect outliers (GSE30999). **(B,C)** Analysis of the scale-free fit index (β) and mean connectivity for various soft-thresholding powers. **(D)** Histogram of connectivity distribution when β = 12. **(E)** Checking the scale-free topology when β = 12. Identification of modules associated with psoriasis.

Supplementary Figure S2
**(A)** Dendrogram of the genes with variance ranked in the top 25% clustered based on a dissimilarity measure. **(B)** Average gene significance and errors in different modules associated with psoriasis. **(C)** Top hub genes in turquoise model selected by the “cytoHubba” plugin in the Cytoscape based on MCC algorithm.

Supplementary Figure S3
**(A,B)** Immunoblotting and analysis of plasma ANGPTL4 of control cream or IMQ treated mice on day 8 (*n* = 6). **(C)** plasma ANGPTL4 level was determined by ELISA in IMQ treatment mice and control cream on day 8 (*n* = 5). Results shown are representative data of three independent experiments. All data were shown as mean ± standard deviation (SD). **p* < 0.05, ***p* < 0.01 vs CON group.
