# Supplementary material for: ANGPTL4 Regulates Psoriasis via Modulating Hyperproliferation and Inflammation of Keratinocytes
Source: Front Pharmacol. 2022 Jul 4;13:850967. doi: 10.3389/fphar.2022.850967 (PMC9289168; doi:10.3389/fphar.2022.850967)
Supplement: Supplementary file 1 [file Table1.docx]

## Supplementary Table S1 : Primer sequences for real-time quantitative PCR.

| **Gene Symbol** | **Forward Primer** | **Reverse Primer** |
| --- | --- | --- |
| Homo IL-1β | ATGATGGCTTATTACAGTGGCAA | GTCGGAGATTCGTAGCTGGA |
| Homo IL-17A | AGATTACTACAACCGATCCACCT | GGGGACAGAGTTCATGTGGTA |
| Homo IL-6 | ACTCACCTCTTCAGAACGAATTG | CCATCTTTGGAAGGTTCAGGTTG |
| Homo TNF-α | CCTCTCTCTAATCAGCCCTCTG | GAGGACCTGGGAGTAGATGAG |
| Homo GAPDH | GGCTCTCCAGAACATCATC | TCTTCCTCTTGTGCTCTTG |
| Mus ANGPTL4 | GGGACTGCCAGGAACTCTTC | GAAGTCCACAGAGCCGTTCA |
| Mus IL-1β | CTTCAGGCAGGCAGTATC | CAGCAGGTTATCATCATCATC |
| Mus IL-6 | CCGCTATGAAGTTCCTCTC | GGTATCCTCTGTGAAGTCTC |
| Mus TNF-α | TGTCCATTCCTGAGTTCTG | GGAGGCAACAAGGTAGAG |
| Mus IL-17A | CTCAGACTACCTCAACCGTTCC | CATGTGGTGGTCCAGCTTTCC |
| Mus GAPDH | TCTCCTGCGACTTCAACA | TGTAGCCGTATTCATTGTCA |
