# Supplementary figures and images for: ANGPTL4 Regulates Psoriasis via Modulating Hyperproliferation and Inflammation of Keratinocytes
Source: Front Pharmacol. 2022 Jul 4;13:850967. doi: 10.3389/fphar.2022.850967 (PMC9289168; doi:10.3389/fphar.2022.850967)

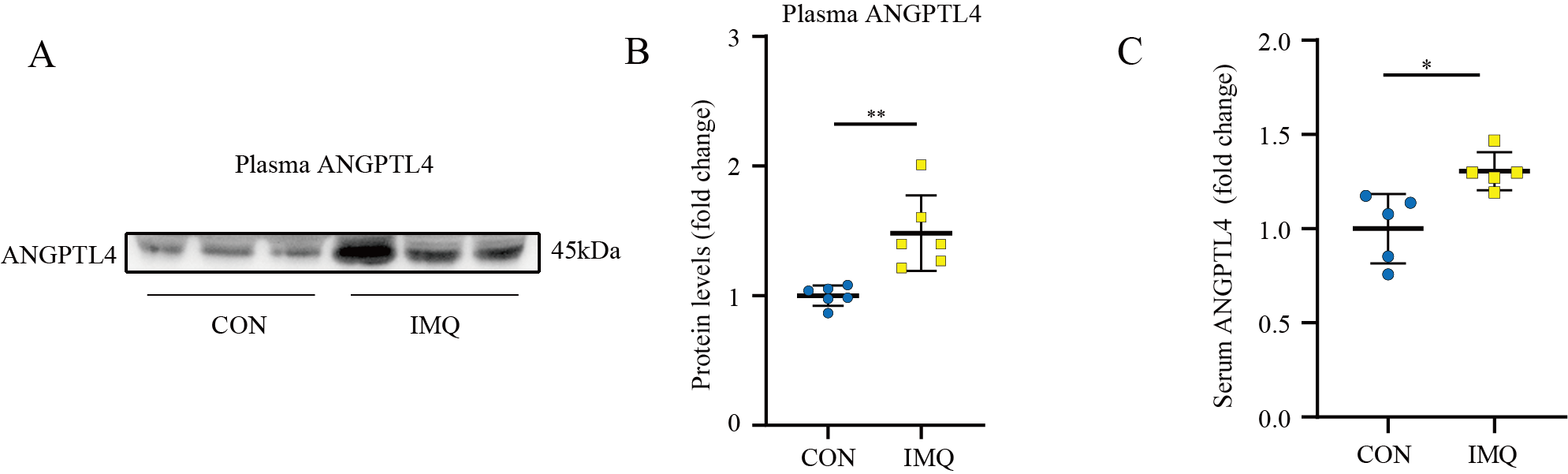

Supplement: Supplementary file 2 [file Image3.tif]

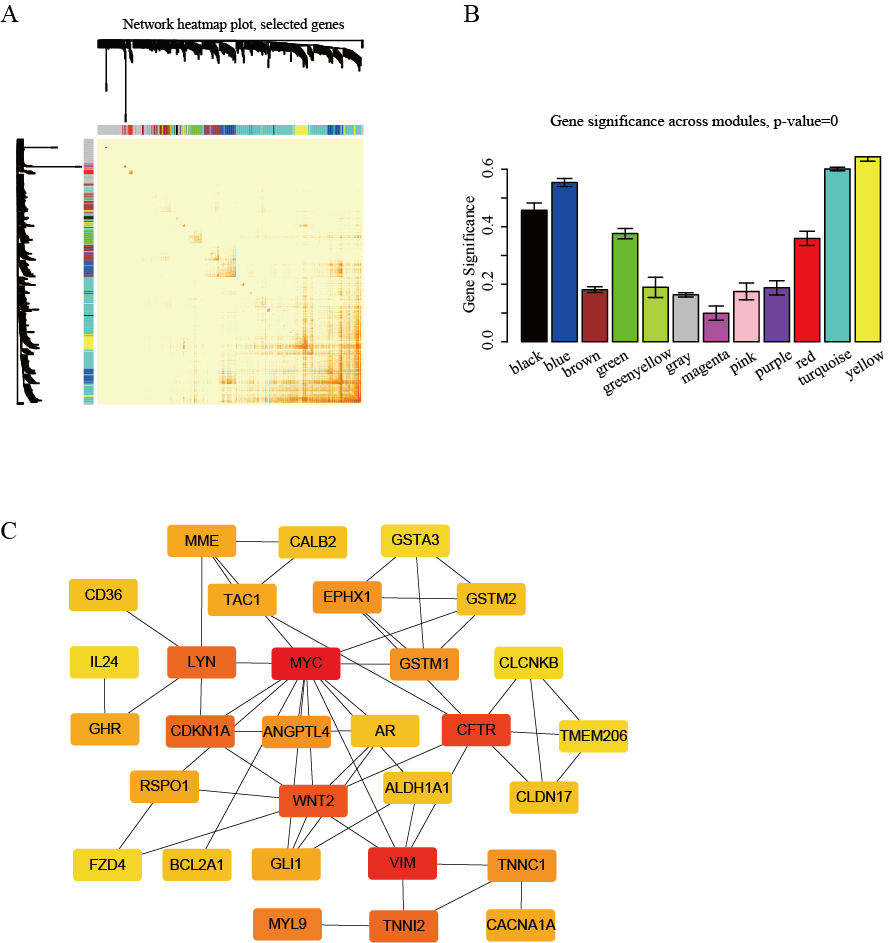

Supplement: Supplementary file 3 [file Image2.tif]

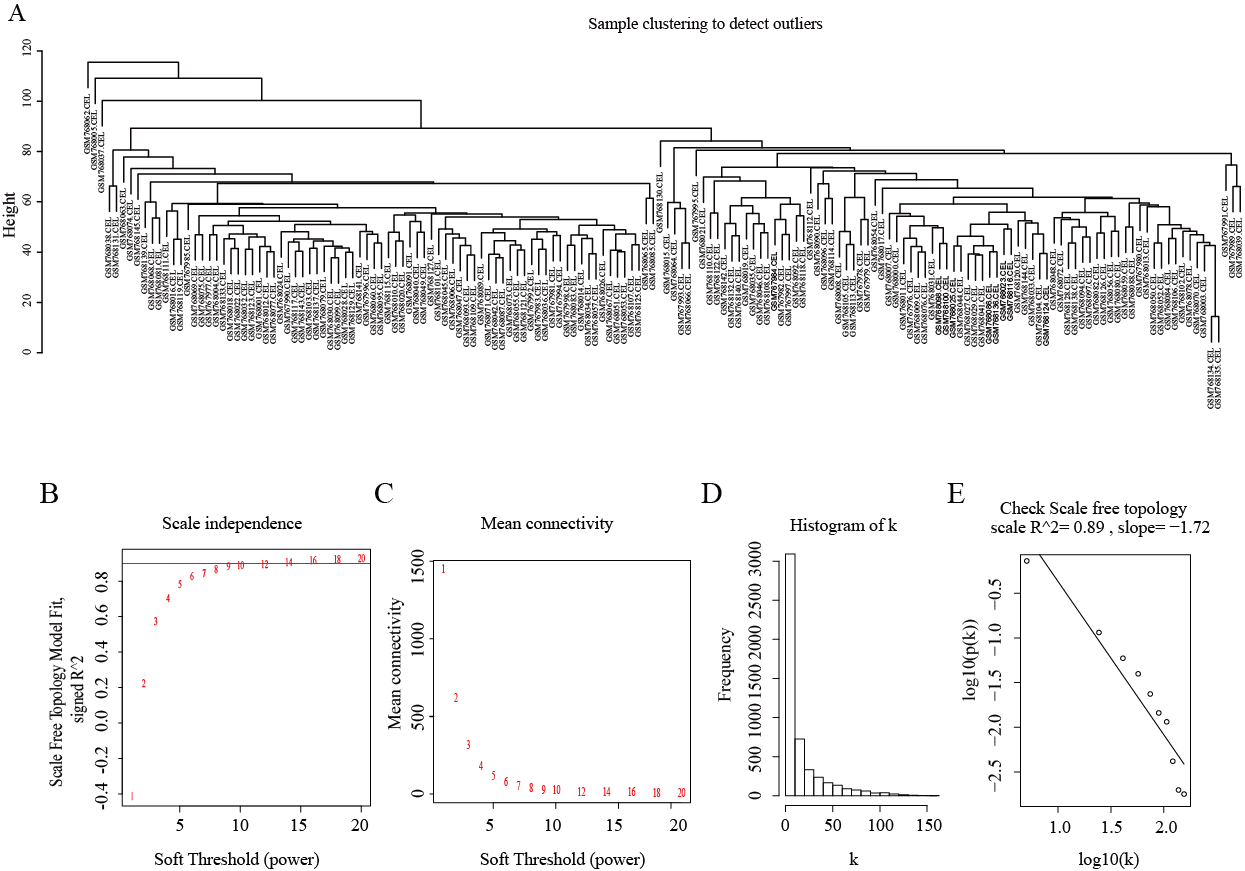

Supplement: Supplementary file 4 [file Image1.tif]
