# Supplementary material for: ANGPTL4 Regulates Psoriasis via Modulating Hyperproliferation and Inflammation of Keratinocytes
Source: Front Pharmacol. 2022 Jul 4;13:850967. doi: 10.3389/fphar.2022.850967 (PMC9289168; doi:10.3389/fphar.2022.850967)
Supplement: Supplementary file 5 [file Table2.docx]

## Supplementary Table S2 : Common genes between the candidate hub genes and DEGs.

| **Gene symbol** | **Full name** | **log_2_FC** | **Adjust *P* value** | **Regulation** |
| --- | --- | --- | --- | --- |
| CXCL8 | C-X-C motif chemokine ligand 8 | 3.88 | 1.26E-41 | Up |
| LCN2 | lipocalin 2 | 3.60 | 3.21E-50 | Up |
| CXCL1 | C-X-C motif chemokine ligand 1 | 3.56 | 1.98E-43 | Up |
| MMP1 | matrix metallopeptidase 1 | 2.80 | 3.16E-26 | Up |
| IL12B | interleukin 12B | 2.77 | 8.13E-36 | Up |
| CXCL10 | C-X-C motif chemokine ligand 10 | 2.51 | 8.84E-28 | Up |
| IL17A | interleukin 17A | 2.47 | 9.56E-44 | Up |
| BCL2A1 | BCL2 related protein A1 | 1.96 | 5.64E-40 | Up |
| CXCL9 | C-X-C motif chemokine ligand 9 | 1.94 | 4.57E-21 | Up |
| IL1B | interleukin 1 beta | 1.90 | 2.32E-29 | Up |
| IL24 | interleukin 24 | 1.59 | 1.48E-24 | Up |
| GABRA4 | gamma-aminobutyric acid type A receptor subunit alpha4 | 1.58 | 1.71E-20 | Up |
| IFNG | interferon gamma | 1.45 | 3.83E-36 | Up |
| LDLR | low density lipoprotein receptor | 1.42 | 1.56E-31 | Up |
| ANGPTL4 | angiopoietin-like 4 | 1.36 | 4.59E-27 | Up |
| LEP | leptin | -1.40 | 6.32E-10 | Down |
| GSTA3 | glutathione S-transferase alpha 3 | -1.71 | 2.36E-23 | Down |
| CCL27 | C-C motif chemokine ligand 27 | -2.13 | 8.04E-32 | Down |
| WIF1 | WNT inhibitory factor 1 | -2.60 | 1.27E-24 | Down |
| CXCL13 | C-X-C motif chemokine ligand 13 | 3.54 | 1.17E-48 | Up |
| CCL20 | C-C motif chemokine ligand 20 | 3.37 | 2.49E-43 | Up |
| GZMB | granzyme B | 3.14 | 3.74E-51 | Up |
| CD274 | CD274 molecule | 2.71 | 2.17E-55 | Up |
| CXCL2 | C-X-C motif chemokine ligand 2 | 2.18 | 6.88E-32 | Up |
| CXCR2 | C-X-C motif chemokine receptor 2 | 2.06 | 3.36E-51 | Up |
| IDO1 | indoleamine 2,3-dioxygenase 1 | 1.92 | 3.2E-41 | Up |
| CTLA4 | cytotoxic T-lymphocyte associated protein 4 | 1.89 | 3.22E-36 | Up |
| CLDN17 | claudin 17 | 1.60 | 1.93E-26 | Up |
| CD80 | CD80 molecule | 1.53 | 1.9E-33 | Up |
| WNT5A | Wnt family member 5A | 1.49 | 4.98E-43 | Up |
| CXCL6 | C-X-C motif chemokine ligand 6 | 1.35 | 5.44E-16 | Up |
| TNNI2 | troponin I2, fast skeletal type | -1.64 | 1.46E-22 | Down |
